# Characterization of *Cronobacter sakazakii* Strains Originating from Plant-Origin Foods Using Comparative Genomic Analyses and Zebrafish Infectivity Studies

**DOI:** 10.3390/microorganisms10071396

**Published:** 2022-07-11

**Authors:** Hyein Jang, Athmanya Eshwar, Angelika Lehner, Jayanthi Gangiredla, Isha R. Patel, Junia Jean-Gilles Beaubrun, Hannah R. Chase, Flavia Negrete, Samantha Finkelstein, Leah M. Weinstein, Katie Ko, Nicole Addy, Laura Ewing, Jungha Woo, Youyoung Lee, Kunho Seo, Ziad Jaradat, Shabarinath Srikumar, Séamus Fanning, Roger Stephan, Ben D. Tall, Gopal R. Gopinath

**Affiliations:** 1 Center for Food Safety and Applied Nutrition, U.S. Food and Drug Administration, Laurel, MD 20708, USA; hyein.jang@fda.hhs.gov (H.J.); jayanthi.gangiredla@fda.hhs.gov (J.G.); isha.patel@fda.hhs.gov (I.R.P.); junia.jean-gillesbeaubrun.civ@mail.mil (J.J.-G.B.); hannahchase11@yahoo.com (H.R.C.); flavianegrete@yahoo.com (F.N.); sfinkel6@terpmail.umd.edu (S.F.); lmweinstein1@gmail.com (L.M.W.); katie.ko@verizon.net (K.K.); nicole.addy3@gmail.com (N.A.); laura.ewing-peeples@fda.hhs.gov (L.E.); junghaa12@gmail.com (J.W.); luy902@naver.com (Y.L.); 2 Institute for Food Safety and Hygiene, University of Zurich, CH-8057 Zurich, Switzerland; athmanya.eshwar@uzh.ch (A.E.); lehnera@fsafety.uzh.ch (A.L.); stephanr@fsafety.uzh.ch (R.S.); 3 Center for One Health, College of Veterinary Medicine, Konkuk University, Seoul 05029, Korea; bracstu3@konkuk.ac.kr; 4 Department of Nutrition and Food Technology, Jordan University of Science and Technology, Irbid 22110, Jordan; jaradatz@just.edu.jo; 5 UCD Centre for Food Safety, School of Public Health, Physiotherapy & Population Science, University College Dublin & WHO Collaborating Centre for *Cronobacter*, Belfield, D04 N2E5 Dublin, Ireland; ssrikumar@uaeu.ac.ae (S.S.); sfanning@ucd.ie (S.F.)

**Keywords:** *Cronobacter sakazakii*, whole genome sequencing (WGS), draft genomes, dried foods, plant-origin foods

## Abstract

*Cronobacter sakazakii* continues to be isolated from ready-to-eat fresh and frozen produce, flours, dairy powders, cereals, nuts, and spices, in addition to the conventional sources of powdered infant formulae (PIF) and PIF production environments. To understand the sequence diversity, phylogenetic relationship, and virulence of *C. sakazakii* originating from plant-origin foods, comparative molecular and genomic analyses, and zebrafish infection (ZI) studies were applied to 88 strains. Whole genome sequences of the strains were generated for detailed bioinformatic analysis. PCR analysis showed that all strains possessed a pESA3-like virulence plasmid similar to reference *C. sakazakii* clinical strain BAA-894. Core genome analysis confirmed a shared genomic backbone with other *C. sakazakii* strains from food, clinical and environmental strains. Emerging nucleotide diversity in these plant-origin strains was highlighted using single nucleotide polymorphic alleles in 2000 core genes. DNA hybridization analyses using a pan-genomic microarray showed that these strains clustered according to sequence types (STs) identified by multi-locus sequence typing (MLST). PHASTER analysis identified 185 intact prophage gene clusters encompassing 22 different prophages, including three intact *Cronobacter* prophages: ENT47670, ENT39118, and phiES15. AMRFinderPlus analysis identified the CSA family class C β-lactamase gene in all strains and a plasmid-borne *mcr-9.1* gene was identified in three strains. ZI studies showed that some plant-origin *C. sakazakii* display virulence comparable to clinical strains. Finding virulent plant-origin *C. sakazakii* possessing significant genomic features of clinically relevant STs suggests that these foods can serve as potential transmission vehicles and supports widening the scope of continued surveillance for this important foodborne pathogen.

## 1. Introduction

The genus *Cronobacter* contains seven species, which include *Cronobacter sakazakii*, *Cronobacter malonaticus*, *Cronobacter turicensis*, *Cronobacter muytjensii*, *Cronobacter universalis, Cronobacter dublinensis* (with three subspecies: *C. dublinensis* subsp. *dublinensis*, *C. dublinensis* subsp. *lausannensis*, and *C. dublinensis* subsp. *lactaridi*), and *Cronobacter condimenti* [1,2,3]. All *Cronobacter* species, except for *C. condimenti*, have been associated with a variety of clinical infections in all age groups. In neonates and infants, the primary illnesses include severe meningitis, septicemia, and necrotizing enterocolitis. In adults, the most common infections include pneumonia, gastroenteritis, septicemia, and urinary tract and wound infections [4,5,6,7,8,9,10]. In fact, according to reports by Patrick et al. [10], Holey et al. [7], Alsonosi et al. [8], and Lepuschitz et al. [11], there are more reported adult infections than there are infantile infections, though the latter are more severe [10,12]. Furthermore, Strysko et al. [12] recently showed that, within the United States, there are higher proportions of infections among full-term and non-hospitalized infants than hospitalized infants. Many of the cases in this report were among infants who consumed powdered infant formula (PIF), reconstituted from opened containers (opened by the infant caretaker in a home) and that this new trend was the predominantly identified transmission vehicle and infection route. At present, there is insufficient epidemiological information to tease apart adult infections originating in a nosocomial setting from those acquired through community-associated sources [13] because most reports document infections of individuals already hospitalized [7,8,10,11]. Of the seven species, *C. sakazakii* has been isolated more often from PIF along with environmental, and clinical samples [14]. Its prominence as a foodborne and public health pathogen was initially elevated to a global concern when contaminated samples of PIF or powdered follow-up formula (FUF) were linked to numerous neonatal meningitis outbreaks [15,16,17,18]. While intrinsically (contamination events occurring during manufacturing processes) or extrinsically (contamination events occurring after manufacturing) contaminated PIF has long been implicated as a vehicle for pathogen transmission in infantile infections, little is known about how adult infections arise [11,12,18].

*Cronobacter* species have been isolated from low-moisture foods like spices, flour, herbs, cereals, and powdered dairy projects in addition to PIF/FUF. Surveillance studies have identified ready-to-eat fresh or frozen produce, dried spices, filth flies, raw materials, and manufacturing plants as additional sources of this pathogen [4,19,20,21,22,23,24,25,26,27,28,29]. Jang et al. [30] demonstrated that *C. sakazakii* strains cultured from filth flies are just as virulent and possess similar sequence types in common with clinical strains. Such strains are receiving more attention, and are now being reported worldwide [23,25,31,32,33]. This increasing body of evidence suggests that plants and plant-origin food may serve as a reservoir or transmission vehicle for *Cronobacter* [29,34,35,36,37,38]. A recent study conducted by Eida et al. [39] reported that *C. muytjensii* JZ38 could colonize plants, providing a benefit to its plant host by sustaining plant growth under salt tolerance stress conditions, while expressing genes involved in the secretion of indole and sulfur-containing volatile compounds. These compounds are thought to play roles in plant growth promotion through increased plant nutrient acquisition and phytohormone production. These results support the original hypothesis conceived by Iversen and Forsythe [4] and by genomic evidence described by Schmid et al. [34], Chase et al. [35], and Grim et al. [40] that a species-level bidirectional evolutionary divergence occurred in the genus which was driven by niche adaptation. However, a better genomic understanding of the prevalence of plant-origin *Cronobacter* strains is needed. Previously, Jang et al. [38] described the genomes of 29 *C. sakazakii* that were obtained from plant-origin foods and dried plant food manufacturing facilities. In another report, the genome features of 26 *C. sakazakii* dried spice strains isolated from the USA, Jordan, and the Republic of South Korea were described by Jang et al. [29]. Interestingly, a metabolic genomic island comprised of a variably sized xylose utilization operon was found within spice-associated *C. sakazakii* genomes which supports the hypothesis that *Cronobacter* species may be able to utilize plant-based carbohydrates as a carbon source and that plants may serve as transmission vectors or substantive hosts [29]. *C. sakazakii* continues to be isolated from various foods of plant-origin (ready-to-eat fresh and frozen produce, flours, cereals, nuts, and spices), which may pose a risk to susceptible consumers [29,34,35,36,37,38]. To date, very little is known about the phylogenomic and virulence traits possessed by such plant-origin *C. sakazakii*. The current study was designed to understand the phylogeny and virulence, using a well-characterized set of *C. sakazakii* originating from plant-origin food, employing molecular, genomic, and pan genomic analyses and zebrafish embryonic infection studies. The finding of strains from plant-origin foods with genomic features similar to clinically relevant and virulent strains of different *C. sakazakii* sequence types (STs) suggests that these foods could serve as potential transmission vehicles and supports widening the scope of continued surveillance of this life-threatening and opportunistic foodborne pathogen.

## 2. Materials and Methods

### 2.1. Bacterial Strains

Thirty-three *C. sakazakii* strains obtained from assorted flours (e.g., corn, rice, soy, unnamed), common food ingredients (e.g., whey powder, sodium caseinate, and seasonings), spices, and other dried plant-origin foods, and manufacturing environments (e.g., USA, Jordan, and South Korea) were investigated in this study. The strains were collected over several years from a variety of plant-origin foods and their production environmental sources from the USA, Republic of Korea, Europe, China, Southeast Asia, and Jordan. The metadata for these strains is shown in Table 1. An additional fifty-five plant-origin *C. sakazakii* strains reported by Jang et al. [29,38] and other relevant *C. sakazakii* were included in this study as needed for comprehensive comparisons and are shown in Supplemental Table S1. All strains were identified as *C. sakazakii* using species-specific (*rpoB* and *cgcA*) PCR assays [41,42,43] and were confirmed using DNA microarray (MA), Multi Locus Sequence Typing (MLST), and whole genome sequencing. O-antigenic serotypes of the strains were determined by following the PCR typing scheme described by Yan et al. [44]. Plasmidotyping was conducted on these strains using primers and PCR assay parameters as described by Franco et al. [45]. Included in the plasmidotyping study were four *C. sakazakii* reference strains: ATCC 29544^T^ Sequence type (ST8), BAA-894 (ST1) [46], GP1999 (ST145) [34,35], and H169/1/16 (ST83) [36].

### 2.2. DNA Extraction from Plant-Origin Isolates

Frozen stocks of each strain were cultured at 37 °C overnight on *Enterobacter sakazakii* Chromogenic Plating Medium (ESPM; R&F Products, Downers Grove, IL, USA). A single colony of each *Cronobacter* strain, displaying the typical blue-black to blue-gray chromogenic colony appearance on ESPM, was inoculated into 5 mL of Trypticase soy broth (BBL, Cockeysville, MD, USA) supplemented with 1% NaCl (TSBS), and then incubated at 37 °C for 18 h with shaking at 150 rpm [29,35,37]. Two-milliliter aliquots of overnight TSBS cultures of each *C. sakazakii* strain were removed and bacterial DNA extraction was performed using a Qiagen QIACube instrument and its automated technology according to the manufacturer’s instructions (QIAGEN Sciences, Germantown, MD, USA). Typical yields of purified genomic DNA were 5–50 µg from a final elution volume of 200 µL. DNA was then used for whole genome sequencing (WGS) and microarray analyses. Each strain’s DNA was quantified using a Qubit dsDNA BR assay kit (Invitrogen, Thermo Fisher Scientific, Wilmington, DE, USA) and Qubit 2.0 fluorometer (Life Technologies, Grand Island, NY, USA).

### 2.3. Whole Genome Sequencing, Assemblies, Annotation, and Multi-Locus Sequence Typing

The extracted DNA samples were prepared for WGS with a molecular biological grade nuclease-free deionized water (Thermo Fisher Scientific, Waltham, MA, USA) with a final concentration of 0.2 ng/μL. Genomic libraries were prepared using a Nextera library preparation kit (Illumina, San Diego, CA, USA) and WGS was conducted using an Illumina MiSeq platform utilizing either 500 or 600 cycles of paired-end reads [29,37,38]. Raw reads (FASTQ datasets) from the Illumina sequencing runs were trimmed for removal of adaptor sequences and for quality control purposes (average read length of paired-end reads ranged from 250 to 300 bases overall). This was followed by de novo assembly using the CLC Genomics Workbench version 11.0 (CLC bio, Aarhus, Denmark). The genomes were submitted to NCBI for PGAP annotation [47] and public release. For verifying annotations, FASTA files of the assemblies were routinely uploaded onto the Rapid Annotation Subsystems Technology (RAST) server (online annotation; http://rast.theseed.org, last accessed 10 November 2021) [48].

Multi-locus sequence typing (MLST) analysis of all strains was carried out by submitting genome sequences (FASTA-formatted files) to the *Cronobacter* MLST website (http://www.pubmlst.org/Cronobacter, last accessed 10 November 2021) where the MLST database assigned allelic, sequence type (ST), and Clonal Complex (CC) profiles for each strain as based on a seven-loci MLST scheme. All genomes were uploaded to the Center for Food Safety and Applied Nutrition’s (CFSAN’s) GalaxyTrakr website for analysis using the server’s bioinformatic toolbox (https://galaxytrakr.org, last accessed 10 November 2021) [49].

### 2.4. Comparative Genome Analyses: Phylogeny, Blast Analyses of Malonate/Xylose Utilization Operons

A core genome analysis workflow originally described by Jang et al. [30] was applied to the WGS datasets in this study. A total of 2790 core genes representing the core-genome of the isolates of plant-origin *Cronobacter* isolates in this study were used to generate a large data matrix to infer phylogenetic relationships. Nucleotide sequences of putative virulence-associated protein-coding gene loci from *C. sakazakii* BAA-894 were used as a reference. Specific genomes were used to generate local databases for customized BLAST analysis using NCBI Blast+ suite [50] analysis as needed. The NCBI standalone BLAST database suite along with in-house Perl and Python scripts (available upon request) were used for SNP detection, processing the data files for output and clustering. The evolutionary distances were calculated with the Maximum Composite Likelihood method, and the phylogenetic tree was built based on the neighbor-joining method as implemented on MEGA X phylogenetic suite [51] downloaded from www.megasoftware.net, last accessed 10 November 2021). The phylogenetic tree was rendered in different formats based on the core gene SNP data-matrix created using the ‘view’ option of the MEGAX suite and a circular format was finally chosen for the lucid representation of the phylogenetic relationships. MLST profiles were used to suggest any simulated lineage groups from near neighbors in phylogenetic trees.

### 2.5. Analyses of Antimicrobial Resistance Genes, Prophage Sequence Profiles, and Clusters of Orthologous Groups

To predict the presence of antimicrobial resistance genes, FASTA formatted genome assemblies were uploaded into the CFSAN GalaxyTrakr AMRFinderPlus tool (https://galaxytrakr.org/, last accessed 10 November 2021) [50] to scan each genome using both the ResFinder 3.1 and PointFinder databases (maintained as webservice by the Center for Genomic Epidemiology, DTU, Lyngby, Denmark). *C. sakazakii* strain 505108 co-harbors three plasmids of different incompatibility classes: IncHI2, IncX3, and IncFIB (virulence plasmid), respectively [52]. FASTA files of plasmid p505108-MDR (National Center for Biotechnology Information [NCBI] accession #: KY978628) and plasmid pNDM (NCBI accession #: KY978629.1) were downloaded from NCBI and used as positive controls for determining antimicrobial resistance genes. The CFSAN GalaxyTrakr’s AMRFinderPlus tool predicted the resistance genes for antibiotics including aminoglycosides; amoxicillin/clavulanate (2:1); ampicillin; chloramphenicol; colistin; cefotaxime; cefoxitin; florfenicol; fusidic acid; gentamicin; macrolides; nitroimidazole; penicillin; quinolone; rifampicin; sulfamethoxazole; spectinomycin; streptomycin; tetracycline; trimethoprim; beta-lactams and extended-spectrum beta-lactams (ESBLs), and ceftiofur, respectively [53]. For prophage sequence identification, plant-origin *C. sakazakii* strain FASTA data sets were uploaded to the PHASTER (PHAge Search Tool Enhanced Release) web server and pipeline (https://phaster.ca/, last accessed 10 November 2021) [54]. Prophage sequences were identified within the contigs of each assembled genome. In addition, EggNOG-mapper analysis for the verification of functional gene annotations and clusters of orthologous groups (COGs) categories was conducted as described by Huerta-Cepas et al. [55], and Jang et al. [29].

### 2.6. Microarray Design, Hybridization, and Analysis

A custom-designed DNA microarray utilizing the whole genome sequences of 15 *Cronobacter* strains, as well as 18 plasmids and developed by the U.S. FDA was used for corroborating WGS-derived phylogenetic characterization of *Cronobacter* species as described by Tall et al. [56]. DNA isolation, fragmentation of the genomic DNA extracted from each strain, hybridization, and processing of the arrays were performed using protocols described previously [30,56,57,58,59]. For each allele represented on the microarray, a fluorescent signal (probe set intensities) was produced and assessed using the Robust Multiarray Averaging (RMA) function in the Affymetrix package of R-Bioconductor as described by Bolstad et al. [60]. RMA summarization, normalization of values, and probe set value determination were carried out as described previously [56,57].

### 2.7. Zebrafish Embryo Infection

To understand the virulence of these plant-origin *C. sakazakii* strains compared to other strains including those of clinical origins, zebrafish infection studies were performed at the University of Zurich, Switzerland. Husbandry, breeding, and microinjection of approximately fifty CFU of bacteria into the yolk sac of 2-day post fertilization (dpf) of the WIK11 zebrafish (*Danio rerio*) line were carried out following the original procedure described in the study by Fehr et al. [61] and as updated by Eshwar et al. [62]. Virulence was assessed by determination of the survival rate (30 embryos: 10 embryos per bacterial strain, in three independent experiments) over 72 hpi (hours post infection = 3 days post-infection). The number of deceased embryos was determined visually based on the absence of a heartbeat. Zebrafish husbandry was conducted with approval (License Number 150) from the Veterinary Office, Public Health Department, Canton of Zurich, Switzerland.

## 3. Results and Discussion

### 3.1. Identities and Genome Properties of C. sakazakii Strains Originating from Plant-Origin Food

**Figure 1:**
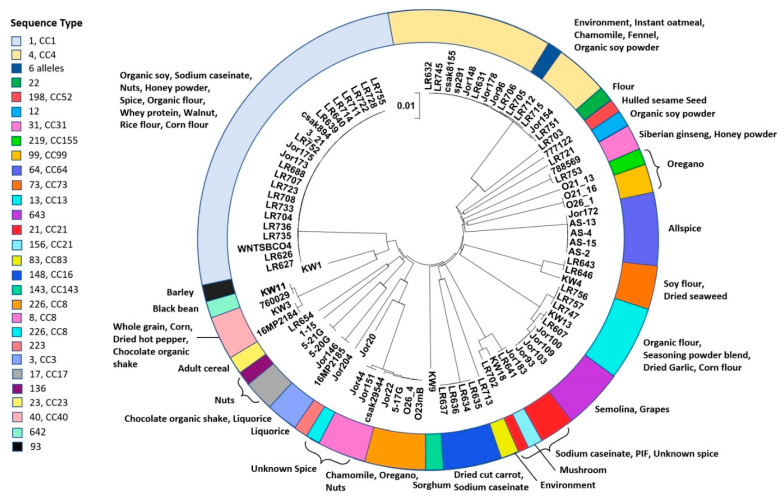
WGS phylogenetic analysis of *C. sakazakii* strains isolated from plant-origin foods and food production environments. A total of 92 genomes from our surveillance (88 plant associated and four reference strains) [13,29,30,35,36,37,38] were analyzed using a new core gene schema reported earlier [30]. *C. sakazakii* strains isolated from plant-origin foods are a highly divergent group of organisms in general. Twenty-nine STs were sorted in multiple divergent groups irrespective of the sample source. The outer ring is color coded that represents sequence type as per the adjacent key. Bar marker represents 0.01 base pair substitutions per site. The tree was built based on a matrix consisting of data-points in 426,143 base positions (spanning 1998 core-gene loci in 92 strains).

Genome properties of the 33 plant-origin *C. sakazakii* strains sequenced during this study are summarized in Table 1. As shown, de novo assemblies of the genomes resulted in an average total genome size of 4505 kb with a range of 4352 to 4679 kb. The average total number of coding regions (CDS) was determined to be 4303 with a CDS range of 4097 to 4540 observed among the genomes. The average GC content of these genomes was 56.8%, with a range of 56.6 to 57.1% being observed. The genome size of the strains from this study are similar to those reported for other *C. sakazakii* strains of plant, food, and clinical origins [29,30,63,64]. Thirteen different STs and at least seven clonal complexes (CC) were found among the plant-origin *C. sakazakii*, including clinically relevant STs such as ST1, CC1 (*n* = 13 strains); ST4, CC4 (4); ST12 (1); ST13, CC13 (3); ST21, CC21 (2); ST64, CC64 (9); ST83, CC83 (1); ST148, CC16 (2); and ST223 (1). Interestingly, LR607 (ST12), a clinical strain without a CC assignment appeared phylogenetically closer to the strain 777122 of ST198/CC52 as seen in Figure 1 (discussed later), and may require further characterization. The MLST designation for strain LR712 matched only six of the seven alleles (*atpD*:5, *glnS*:3, *gltB*:3, *gyrB*:5, *infB*:5, *pps*:4) which were indistinguishable from the members of CC4; its *fusA* allele sequence was not identified by MLST analysis. This result suggests that this *C. sakazakii* strain may possess a novel ST designation which phylogenetically is related to members of CC4. Five different serotypes were represented in these strains including CsakO:1-CsakO:4, and CsakO:7. One strain’s serotype (KW9 isolated from the cereal plant sorghum, Republic of Korea) could not be determined. Genotypic characterization of markers followed the pattern reported in other studies [65,66]. All assemblies were released under FDA GenomeTrakr Bioprojects on NCBI (PRJNA258403 and PRJNA613494) which are part of the FDA Center for Food Safety and Applied Nutrition (CFSAN) foodborne pathogen research umbrella project at NCBI (PRJNA186875). Contigs containing sequences homologous to the virulence plasmid pESA3 from *C. sakazakii* BAA-894 reference strain were found in the WGS assemblies of all plant-origin *C. sakazakii* strains, and were confirmed by conventional PCR based on Franco et al. [45].

### 3.2. Zebrafish Embryo Infectivity Studies

**Figure 2:**
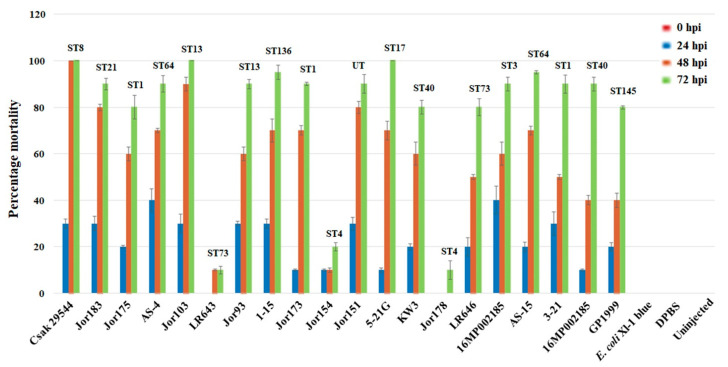
Results of zebrafish embryo infection (ZI) experiments (time course 0–72 hpi (hours post infection)) with plant-origin and malonate-positive *C. sakazakii* ST64 strains compared to clinical strain ATCC 29544^T^ (ST8) and *E. coli* Xl-1 blue as positive and negative control, respectively. DPBS indicates Dubelcco’s phosphate-buffered saline. Jor103 and 5-21G of the 19 *C. sakazakii* strains killed 100% of the zebrafish embryos in 72 h in a similar manner to *C. sakazakii* strain ATCC 29544^T^. Except for three strains, the other 14 strains killed at least 80% of the zebrafish embryos by 72 hpi. Three plant-associated strains, LR643 [ST73], Jor154 [ST4] and Jor178 [ST4], had mortality less than 20% in this time-period.

Nineteen *C. sakazakii* strains from plant-origin foods were tested in the zebrafish embryo model and compared with *C. sakazakii* clinical strain ATCC 29544^T^ and *E. coli* Xl-1 blue which were included as positive and negative controls, respectively. As shown in Figure 2, two (Jor103, 5-21G) of the 19 *C. sakazakii* strains killed 100% of the zebrafish embryos (mortality measured by 72 hpi) in a similar fashion as that of *C. sakazakii* strain ATCC 29544^T^. Except for three strains (LR643 [ST73], Jor154 [ST4], Jor178 [ST4]), the other 14 strains killed at least 80% of the zebrafish embryos by 72 hpi. These results suggest plant-origin strains are as virulent as clinical strains in this model system, but there are differences in strains due to the presence of additional unknown factors contributing to pathogenicity [62].

### 3.3. Phylogenetic Characterization of Cronobacter Strains from Plant-Origin Foods Using a Genus-Specific Core-Gene MLST Schema

In addition to the 33 strains in Table 1, additional genomes from other plant-origin *C. sakazakii* previously reported [29,38] as part of our environmental surveillance effort were co-analyzed in a comprehensive manner (Supplemental Table S1). Whole genome sequence diversity had been already used by our group to study strain-level differences in *Cronobacter* with different methods [36,56]. A systematic bioinformatic approach using selected core-gene loci spanning the whole genome to understand the phylogeny of *Cronobacter* species was developed [30]. We applied this core-gene scheme consisting of 2000 loci to the plant-origin isolate genomes from this study to determine the extent of sequence diversity in terms of single nucleotide polymorphisms (SNPs) among these closely related *C. sakazakii*. Ninety-two genomes from this dataset including the 88 plant-origin strains were analyzed by first creating a local BLAST database as described in Section 2.7 and identifying SNPs in each of the gene loci queried against the database. A large data matrix made up of polymorphic sites in each of the 92 strains spanning 1998 genes (derived from ~426,000 × 92 data points) was generated from this SNP-finding workflow (to be shared on request). The SNP profiles of the queried strains were aligned for calculating a distance matrix as described earlier. A Neighbor-Joining phylogenetic tree was built on this data matrix to cluster closely related strains and to distinguish phylogenetically related clusters of *C. sakazakii* strains based on their SNP characteristics in the 1998 core gene loci (Figure 1). The core-genome-based approach implemented on the *C. sakazakii* strains from diverse sample sources was able to capture the intra-species diversity and illustrate emerging lineages.

In this phylogenomic analysis, the plant-origin strains were clustered following their respective STs (see Figure 1) as expected. The predominant ST1 cluster included strains from dried food sources like organic soy and other flour types as well as clinical strains like BAA-894. A similar result was observed for the ST4 strain cluster which consisted of organisms isolated from clinical, spice, and environmental sources. Previous studies identified that spices constitute a major source for *C. sakazakii* strains of many ST groups. For example, Jang et al. [29,38], Jaradat et al. [65], and Chon et al. [66] have observed a similar diversity of *C. sakazakii* associated with a variety of spices and herbs. The diversity of plant-origin *C. sakazakii* observed from the core-gene analysis in this study showed that these strains phylogenetically clustered into 29 different ST groups (Figure 1). There was no direct correlation observed between the STs and the sample sources suggesting a broader prevalence in plant-origin food sources for *C. sakazakii.*

To investigate emerging genotypic variations in core genome loci among the 88 plant-origin *C. sakazakii* strains, a smaller slice of the initial SNP matrix was analyzed. Some previously reported clinical genomes (e.g., ATCC 29544^T^, 8155, BAA-894) and 38 strains selected from our surveillance project belonging to 25 STs were included in this analysis. We attempted to simulate ‘lineage groups’ to refer to a shared evolutionary lineage (Supplemental Figure S1) when strains of different STs with overlapping MLST alleles co-clustered or when plant-origin strains of the same ST grouped together with known clinical isolates—both cases reflecting their conserved core-gene sequence features. Some of the suggested lineage groups that included clinical strains were monotypic like those belonging to ST1, ST4, and ST8. Another two lineage groups consisting of ST40, ST642, and ST13, ST643, respectively, included only plant-origin strains in this illustration. This simulated grouping of strains of different STs clustered into apparent lineage groups as seen in Supplemental Figure S1 and highlights the emerging nucleotide divergence among plant-origin *C. sakazakii* observed in this study, and it is reflective of the diverse genotypes observed among *C*. *sakazakii* in general. Most importantly, this brings out the sharing of core genomic backbone among the plant-origin and clinical strains. For example, this emerging genotype miscellany is reflective of the diversity recognized in strains from global sources such as those seen in the *C. sakazakii* genome tree from the NCBI Genome database (https://www.ncbi.nlm.nih.gov/genome/tree/1170?, last accessed 10 November 2021). The easy availability of large numbers of WGS datasets and consequent extensive phylogenetic analysis of various pathogens have repeatedly highlighted emerging properties of transmission to new hosts, in response to changes in environment or antimicrobial stress, and rapid niche adaptation. This information provides some insights into developing analytical methods based on genetic determinants associated with these emerging properties [67]. The underlying molecular basis for the simulated lineage groups described above could be further evaluated in high resolution by data-mining the large SNP data matrix described earlier. This is important for understanding the actual sequence diversity and the extent of shared core-genome genotypic characteristics that could define a lineage group. Any shared microevolutionary processes reflected by related SNP profiles at a genome-level rather than just seven MLST markers would be more informative and accurate in defining ‘lineage groups’ in *C. sakazakii.* Based on our surveillance of plant-origin *Cronobacter* strains, it is evident that a phylogenomic analysis of global collections of the organisms of this genus will be essential to understand their emerging genotypic diversity and their prevalence in hitherto unidentified environmental sources.

**Figure 3:**
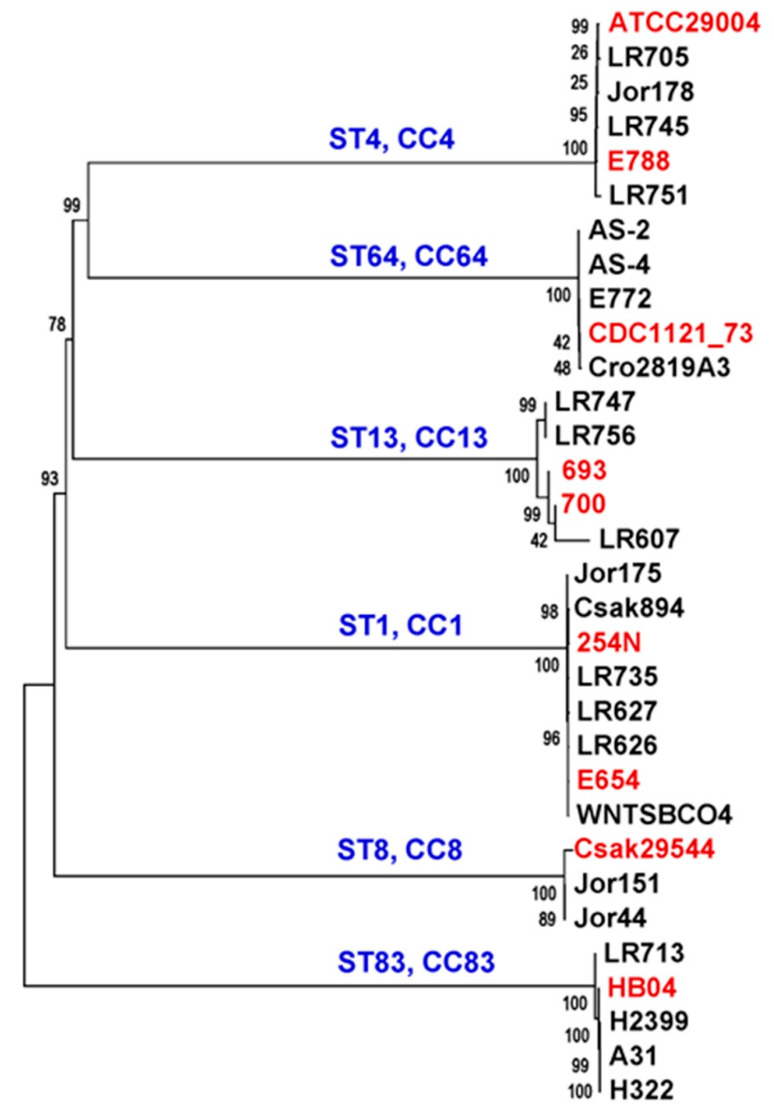
Co-clustering of plant-origin strains with clinical *C. sakazakii* genomes. Information of genomic properties of clinical strains used in the analysis is available in Supplemental Table S1. The initial allele matrix was used to identify clinical near-neighbors of plant-associated *C. sakazakii*. A representative group of isolates belonging to six STs is given where the red color indicates clinical strains. Blue font indicates ST and CC lineages.

*C. sakazakii* strains from various STs such as ST1, ST4, ST8, ST13, ST64, and ST83 have been reported to cause illness through suspected contamination of PIF and other products [13]. Clinically relevant strains constituted multi-type lineage groups along with plant-origin strains as inferred from Supplemental Figure S1, which suggests a shared conserved core-genome backbone profile. To analyze this pattern in high resolution, a data matrix of core-gene SNPs from the genomes of a short list of plant-origin strains and representative clinical strains was generated, and a phylogenetic tree was built. Predictably, plant-origin and clinical strains co-clustered according to their ST types (Figure 3), where the clinical strains are shown in red. PIF and a small number of strains from dried dairy powder products have been historically implicated in *Cronobacter* pathogenesis in neonatal and immunocompromised patients. Co-clusters of novel plant-origin strains with known clinical strains based on conserved core-genome profiles would allow us to bioinformatically predict clinically relevant attributes in these environmental strains that are poised in proximity for potential entry into the human food supply. For example, homologs of the virulence plasmid pESA3 (from *C. sakazakii* BAA-894 in the ST1 cluster, Figure 3) were also seen in co-clustering plant-origin strains from this pilot study. Additionally, ST83 strains tightly cluster together with environmental strain LR713 in this study, grouping with previously reported clinical strain HB04 and three PIF strains, A31, H322 and H2399 (Figure 3; Supplemental Table S1). Our observations from this preliminary predictive analysis of co-clustering strains from clinical and food sample sources should enable future detailed genotype-phenotype studies to be carried out using functional genetic approaches designed to characterize pathogenic genotypic features. In fact, some plant-origin strains from this study form a multi-sequence type ‘lineage group’ (Supplemental Figure S1) like 1–15 (ST136), 5-21G (ST17), and a ST3 (16MP002185) strain appear to display significant virulence in a zebrafish embryo model (Figure 2). Lineage groups with co-clustering strains belonging to ST1 (Figure 3) with three known clinical isolates (Csak894, 254N and E654), two spice isolates from this study Jor173, Jor175, and 3_21(nuts) were highly virulent to zebrafish embryo in the bioassay (Figure 2). Different factors probably contribute to the clinical pathogenicity and virulence potential of strains even when sharing genotypic profiles. This resulted in some co-clustering strains from the same lineage group displaying different phenotypes. For example, in the lineage group with ST8 co-clustering isolates, a spice isolate Jor151A, was as virulent in the assay as the clinical strain ATCC 29544^T^. Two spice strains of monotypic lineage group ST4, Jor154 and Jor178 (Supplemental Figure S1) appear to be avirulent in the bioassay (Figure 2) though co-clustering with clinical strains ATCC 29004 and E788 (Figure 3).

This de novo core-genome analysis of *C. sakazakii* strains from a variety of sample sources opens a new venue for comparative genomics of this emerging pathogen. This genome-wide approach suggests that global nucleotide diversification of these emerging pathogens is differentially impacted by the niches they occupy even when they share a ST profile and persist in the human food supply. The efficacy of clustering *C. sakazakii* strains as lineage groups based on a shared core genome backbone must be further studied using extended surveillance, additional WGS datasets with detailed sample metadata, and functional genetics studies. Such a comprehensive and systematic approach may lead to a better understanding of genetic determinants of pathogenicity, predicted or tested, in *Cronobacter* strains that are prevalent and associated with plants and plant-origin products that are also shared by components of the human food supply.

### 3.4. Identification of Antimicrobial Resistance Genes, Prophage Signature Sequences, and Clusters of Orthologous Groups in Plant-Origin Cronobacter Strains

*(a) Antimicrobial Resistance Genes:* When analyzed with AMRFinderPlus [53] implemented on CFSAN’s GalaxyTrakr [49], all 88 *C. sakazakii* strains from plant-origin foods were found to possess a CSA family class C β-lactamase gene, *bla*_CSA_ (subclass of cephalothinases) (Supplemental Table S2). As shown in Table 2, two of the strains (Jor22 and Jor20) were identified as possessing the *bla*_CSA-1_ variant gene, while 12 strains (LR631, LR632, LR705, LR706, LR712, LR715, LR745, LR751, Jor96, Jor148, Jor154, and Jor178) possessed a *bla*_CSA-2_ variant gene as described by Müller et al. [68] and the other 74 strains were shown to possess *blacsa* (Table 2). In addition to identifying the presence of *bla_CSA_* resistance genes in the strains, *C. sakazakii* strains LR735, LR736, and WNTSBOCO4 also possessed a *mcr-9.1* colistin resistance gene (encoding for phosphoethanolamine-lipid A transferase) (Table 2). Interestingly, in both LR736 and WNTSBOCO4, the *mcr-9.1* colistin resistance gene (WP001572373.1) was adjacent to an IS5 insertion element (IS5 family transposase, WP001572374.1).

The cationic polypeptide antibiotic colistin belongs to the group of polymyxins that has been used for decades in animal production facilities for the treatment of infections caused by *Salmonella enterica* subspecies *enterica* and *Escherichia coli* that are associated with the husbandry of pig, cattle, sheep, and poultry food animal species [69]. Of great clinical concern is the rapid spread of *mcr*-resistance genes and carbapenem-resistance genes among *Enterobacteriaceae* resulting in the emergence of true pan-drug resistance and is evidence of microevolution mechanisms occurring with plasmid attributes. The presence of the colistin resistance gene, *mcr-1* in *E. coli* and *Cronobacter* strains in China was first reported by Lui et al. [70]. This AMR gene was found to be carried on an IncI2-like conjugative plasmid, pWF-5-19C (65,203 bp in size). This was quickly followed by numerous studies demonstrating the spread of *mcr-1* to several *Enterobacteriaceae* species as well as to multiple continents [71,72,73,74]. Recently, Yang et al. [75] presented evidence that the colistin resistance gene, *mcr-10* was being carried on the *C. sakazakii*-like virulence plasmid (pCsaCS931b) described by Yang et al. [75]. Little is known about the role of produce in the transmission of AMR genes, but Hölzel et al. [76] provide a summary of the literature which revealed that vegetables may potentially, although rarely, be a vector for the transmission of AMR genes to humans and other animals. This study emphasized the fact that many vegetables are consumed raw and that immunocompromised individuals should be aware that they are predisposing themselves to possibly antibiotic resistant organisms. Finally, a report by Shi et al. [52] demonstrated the extent to which antibiotic resistance in *C. sakazakii* has become a global problem, in that in only two years these authors reported two conjugative plasmids (p505108-MDR and p505108-NDM) carrying 21 different drug resistance genes in a ST1 *C. sakazakii* (strain 505108) including the *mrc-9.1* colistin resistance gene.

*(b) Prophage Signature Sequences:* Prophages comprise a major genomic portion of bacterial communities in most ecosystems, and they play important roles in virulence and evolution of important bacterial pathogens [77]. DNA acquired through horizontal gene transfer (HGT) mechanisms such as through the acquisition of prophages is a primary driving force in the evolution of bacteria [78] and contribute to genomic differences among bacterial species. A few prophage sequences have been observed in the genomes of *Cronobacter* strains. Three putative unnamed prophage genomes and three other incomplete genomic encoding regions in *C. sakazakii* BAA-894 were identified by BLAST analysis [46] in the Tennessee neonatal intensive care outbreak strain [15]. Additionally, in 2012, three complete lysogenic *Cronobacter* bacteriophage genomes (ENT47670, ENT39118, and phiES15) were reported [79,80]. To understand the complexity and diversity of prophage genomes within the 88 plant-origin *C. sakazakii* strains, the PHASTER bioinformatic web server tool was used [54]. PHASTER analysis identified 363 incomplete and intact prophage genomic encoding regions within these strains isolated from plant-origin foods. It identified 185 intact phages among the strains including the three intact and previously described *Cronobacter* phages, ENT47670, ENT39118, and phiES15 (Supplemental Table S3). Five strains carried two of the three *Cronobacter* phages either in combinations of ENT39118 and ENT47670 (four strains), and ENT39118 with phiES15 in one strain. There were considerable size differences noted among these phage genomic regions which ranged from 30.8 to 59 Kbp in size. Interestingly, PHASTER identified between 3 to 28 genes encoding for hypothetical proteins in these three prophage genomic regions which supports the prophage contributions reported by Zeng et al. [77]. Among the strains, PHASTER analysis identified 22 different intact prophage encoding regions that presumably arose through transduction of phage from eight different bacterial species. These intact phage encoding regions spanned from 17.2 to 115 kbp in size and encoded for 21 to 142 total proteins. The diversity of phages possessed by these strains is shown in Supplemental Table S3 (see ‘Most Common Phage hit’ with ‘intact’ of completeness). A PhiO18P phage (NC_009542) from *Aeromonas* was identified in *C. sakazakii* LR721 and seven different *Salmonella* prophages were identified in fifty-nine of the *C. sakazakii* strains (e.g., *Salmonella* RE2010, NC019488; *Salmonella* 118970sal3, NC031940; *Salmonella* SSU5, NC018843 to name a few). Of critical note, Negrete et al. [81] recently described the integration of *Salmonella* SSU5 prophages within pCS1, pH322_1 and pGK1025B_1 like plasmids. Furthermore, subsequent closure of these genomes will help to support these findings. Two different *Shigella* prophages were also identified in the *Cronobacter* genomes. *Shigella* SFII (NC021857) prophage was found in strains 5-20G and KW4, and *Shigella* Sf6 (NC005344) prophage was found in LR634 and LR635. Four different *Enterobacter* prophages were identified in ten strains. *Enterobacter* mEp235 (NC019708) was found in two strains (KW18, LR702) while both *Enterobacter* HK542 (NC019769) and *Enterobacter* 186 (NC001317) each were found in 5–17G and Jor204, respectively. *Enterobacter* ES18 (NC006949) was identified in six strains (e.g., LR639, LR711, LR714 to name three). Together, these results demonstrate the complexity and the representative total genomic content of lysogenic phage sequences in these strains isolated from plant-origin foods.

*(c) Clusters of Orthologous Groups:* To understand the cellular functions possessed by plant-origin *C. sakazakii* strains, the genomes were also analyzed using the eggNOG database to assign Clusters of Orthologous Groups (COG) functional categories to the various set of genes [55]. Average numbers of protein-coding genes associated with general COG functional categories [82,83] are presented in Table 3 and information for individual strains is shown in Supplementary Table S4. The COG category S for uncharacterized proteins had the highest number (27.3%) of assigned genes. The overall distribution of the average percentage of each COG category (Table 3) showed a similar pattern of the COG functional analysis as that of a previous study which described genomic properties of *C. sakazakii* strains obtained from dried spices [29]. In summary, genes involved in carbohydrate transport/metabolism, transcription, and amino acid transport/metabolism were found to be the most abundant in these plant-origin *C. sakazakii* strains. The high abundance of these genes in these strains suggests that they function to help with the interaction between microorganisms and their plant host such as maintenance of the cellular machinery involved in facilitating the growth and persistence of *C. sakazakii* within these diverse and complex microbial communities. The *C. sakazakii* genomes from this study possessed a large proportion of putative proteins (~50% of assigned COG categories) associated with transport and metabolism of carbohydrate, amino acid, inorganic ion, coenzyme, cell wall/membrane biogenesis, and posttranslational modification/protein turnover and could contribute to the pathogen’s survival within the plant associated osmotically-challenging eco-niche [15,29,35,36,37,38,39,84,85,86,87].

### 3.5. Prevalence and Distribution of Plasmids and Respective Virulence Factor Genes among C. sakazakii Strains Derived from Plant-Origin Foods

The prevalence and distribution of plasmids pESA3, CSK29544_1p, pCS2, pGW2, p1CFSAN068773, pSP291-1, pESA2, and pCTU3-like plasmids and their plasmid-borne genes were determined by PCR as described by Franco et al. [45]. In this study, 88 plant-associated *C. sakazakii* strains and four control strains (ATCC 29544^T^, BAA-894, GP1999, and H169/1/16) were examined and the results are shown in Table 4. In total, all 92 strains possessed the pESA3-like virulence plasmid. However, only 5 of the 92 strains possessed the conjugative pESA2-/pCTU2-like plasmid, and 20 of the strains possessed the pCTU3-like plasmid. All strains isolated from plant-origin foods possessed *cpa* (*Cronobacter* plasminogen activator gene encoding outer membrane protease), and the two iron acquisition systems (ABC transporter gene cluster: *eitCBAD* and a hydroxymate aerobactin-like siderophore gene cluster: *iucABCD/iutA*, named *Cronobactin*) contained on the pESA3-like virulence plasmid, similar to what others have reported [29,37,38,45,81]. Interestingly, in Table 4, all but one strain possessed the *intL* gene target, representing the left side of the type 6 secretion system (T6SS), while 50, 56, and 29 of the 92 strains possessed *vgrG*, *T6SSRend*, and *IntRT6SS* gene targets, respectively, and the significance of which supports the results of others which suggest that this region is in considerable genetic flux [13,45,79,86]. Lastly, 19 of the 92 strains possessed the *Bordetella pertussis*-like *fhaB* gene target, which represents the filamentous hemagglutinin gene cluster described by Franco et al. [45]. It was interesting to find that more strains possessed the pCTU3-like plasmid rather than the conjugative pESA2/pCTU2-like plasmids. Other studies have reported similar results [45]. pCTU3-like plasmids which possess a plethora of heavy metal-related efflux pumps such as *cusA/czcA* heavy metal efflux RND transporter system [88] may be important in adapting the organism to changing osmotic growth conditions (stress) and its persistence as a member of the plant associated eukaryotic microflora. A more comprehensive analysis including candidates of closely related *Enterobacteriaceae* members may shed more light on the acquisition and functionality of heavy metal efflux pump gene systems in *Cronobacter*.

### 3.6. Prevalence and Distribution of the Xylose Utilization Operon in Cronobacter Strains Isolated from Plant-Origin Foods

The xylose utilization operon in the *Cronobacter* strains from this study consisted of homologous genes found in *E. coli* K-12 strain MG1655). Additionally, the variable lengths of these operons (ranging from 16,340 to 16,790 bp) with either truncated or duplicated *bax* and the *α-xynT* genes and other genotypic features, were comparable to a previous study by Jang et al. [38]. In another study, Jang et al. [29] extended these observations by defining the xylose utilization operon as a metabolic island which was originally described in spice associated *C. sakazakii* genomes. Our analyses of the 88 strains, which included the strains from these two studies [29,38], isolated from plant-origin foods generally support the hypothesis that plants may be the ancestral eco-niche for *Cronobacter* and that the xylose utilization operon might contribute to its adaptation and survival as posited by Schmid et al. [34], Joseph et al. [14], and Chase et al. [35].

### 3.7. DNA Tiling Microarray Analysis

Microarray analysis of 88 plant-origin and four reference strains of *C. sakazakii* using a pan-genome DNA tiling array platform is shown in Supplemental Figure S2. The dendrogram based on distance metrics was first developed using the presence/absence calls of ~19,000 genes captured on the microarray and then MLST data obtained from the *Cronobacter* MLST web site (https://pubmlst.org/Cronobacter/, last accessed 10 November 2021) was overlaid onto the strains within the resulting cluster diagram. Three major clusters were identified, and the strains grouped according to sequence type designations (Supplemental Figure S2). The first major cluster consisted of 22 ST1, CC1 strains which came from PIF samples (control *C. sakazakii* strain BAA-894), various dried flours, nuts, sodium caseinate, honey powder, and spices. The diversity was represented by two subclades. The second major cluster was made up of five smaller clades each with strains with defined STs such as ST4, CC4; ST31, CC31; ST148, CC16; ST3, CC3; ST17, CC17; ST99, CC99; ST73, CC73; ST64, CC64; ST40, CC40; and ST13, CC13, respectively. There were an additional eleven strains (singletons) possessing STs of ST219, CC155; ST22; ST218; ST93; ST223; ST145; ST136; ST23, CC23; ST198, CC52; and ST12. Interestingly, one ST13, CC13 strain (LR747) clustered with a group of 11 ST4, CC4 strains and a single ST4, CC4 strain (LR745) clustered with eight ST13, CC13 strains. These results support the hypothesis that MA analyses using the over 19,000 *Cronobacter* genes captured on the microarray is more resolving than the seven-allele MLST scheme. This was first noted by Chase et al. [36] and supported by the results reported by Jang et al. [13]. The last major cluster consisted of strains possessing the following ST allelic profiles: ST8, CC8; ST226, CC8; ST156, CC21; and ST121, CC21. There was a single ST1, CC1 strain that clustered with the ST156, CC21 and ST121, CC21 strains. These data also support the clonal complex hypothesis first reported by Joseph et al. [14] which showed that strains possessing phylogenetically related STs form a group of highly related strains in a clonal complex. Another interesting finding is the clonal relatedness among *C. sakazakii* strains possessing the ST73, CC73; ST145; and ST64, CC64 allelic profiles. The ST145 strain is represented by GP1999, which is an isolate obtained by Schmid et al. [34] from a tomato plant. It should be emphasized that MA demonstrates the relatedness of the plant-origin *C. sakazakii* strains possessing ST allelic profiles in common with clinically relevant *C. sakazakii* strains.

These results from the *Cronobacter*-specific microarray analysis based on nucleotide divergence inferred from the hybridization profiles of DNA from the samples (Supplemental Figure S2) corroborate the phylogenetic relationship among the plant-origin *C. sakazakii* isolates elucidated by the core-genome analysis (Figure 1) involving SNPs identified from the homologs of about 2000 core gene loci in their genomes. Data mining of the microarray database maintained within our laboratory allowed us to compare the nucleotide profiles and conserved mobilome of the 88 plant-origin *C. sakazakii* strains with that of hundreds of legacy strains in our collection (personal communication, BDT). As expected, the DNA tiling microarray data analysis captured known genomic features in the new strains. Nevertheless, WGS-based analysis alone clearly highlighted the emerging sub-groups and their nucleotide diversity within this clinically important foodborne pathogenic species. Elucidation of new genotypic features like new plasmids [80] and new insights into phylogenetic properties of sub-clades of *C. sakazakii* lineages observed in this study only when WGS data was used for analysis. This reiterates the necessity of evaluating the application of our core-gene schema to carry out routine analysis of *Cronobacter* strain collections from surveillance projects or foodborne outbreak investigations.

### 3.8. Important Lessons from This Surveillance Study for Food Safety

*Cronobacter* species have undergone a significant reclassification [1,2] and the genus currently has seven species; undoubtedly, this taxonomic scheme will continue to evolve [3]. Once thought to be only a harmless inhabitant of the intestinal tract of humans, *Cronobacter* species are now considered to be a group of pathogens with notable versatility in their ability to cause human disease in all age groups [13]. Since the late 1990s, a lot of emphasis has been focused on controlling the organism in food products such as PIF that is consumed by infants. This is rightfully a very important endeavor because of the severity of infections and loss of life that this organism has caused in this very susceptible population. However, epidemiologically, studies reported by Iversen and Forsythe [4], Patrick et al. [10], Holy et al. [7], and Alsonosi et al. [8] and others have shown that *Cronobacter* species cause a higher number of infections in adults. Furthermore, surveillance studies have shown that *C. sakazakii* can also prevail in food manufacturing environments (built environments) such as PIF manufacturing facilities, and the farm to fork continuum with potential to cross contaminate foods posing a risk to susceptible consumers [27,87]. Many previous reports on finding *Cronobacter* contamination also in plant-origin food sources in addition to PIF were confirmed by our results as shown. It has thus become imperative to understand the genomic diversity of *C. sakazakii* strains associated with plant-origin food sources for better food-safety policy and practice.

## 4. Conclusions

The results collected in this study increase the number of publicly available *Cronobacter* genomes, including other malonate-positive *C. sakazakii* strains isolated from food and clinical sources, which possess sequence types other than ST64. These data also further support the establishment of the zebrafish embryo infection model, and its ability to play a key role in high throughput comparative genomics experiments, to help unveil the virulence determinants of *Cronobacter* species that contribute to human disease. Our findings indicate that the plant-origin *C. sakazakii* strains (i) could harbor genomic features like AMR genes; (ii) are capable of virulence in a zebrafish infectivity model similar to strains of clinically relevant STs; and (iii) may share conserved core genome SNP profiles with genomes of clinical strains. These observations strongly suggest that these foods could serve as potential transmission vehicles and support widening the scope of continued surveillance of this important foodborne pathogen. It is critical to note that the host range and sample sources of *C. sakazakii*, long considered a pathogen connected with few foods like PIF, are increasing and future surveillance studies are warranted to understand the extent of its global prevalence.

## Figures and Tables

**Table 1:** Strain name, source, country of origin, genome size, G + C% content, number of Coding DNA sequences (CDS), sequence type (ST), serotype, and NCBI GenBank accession numbers of 33 *C. sakazakii* isolates obtained from plant-origin foods and sequenced in this study.

Strain	Source	Country	Genome Size (kb)	G + C Contents (%)	No. of CDSs	ST ^a^, CC ^b^	Serotype ^f^	NCBI Accession No.
MOD1_LR607	Corn flour	USA	4577	56.8	4399	13, CC13	CsakO:4	PVCO00000000
MOD1_LR626	Rice flour	USA	4547	56.8	4345	1, CC1	CsakO:1	PVCR00000000
MOD1_LR627	Corn flour	USA	4405	56.8	4204	1, CC1	CsakO:1	PVCM00000000
MOD1_LR636	Sodium caseinate	USA	4420	57	4177	148, CC16	CsakO:1	PVCN00000000
MOD1_LR637	Sodium caseinate	USA	4421	57	4176	148, CC16	CsakO:1	PVCS00000000
MOD1_LR639	Sodium caseinate	USA	4557	56.7	4365	1, CC1	CsakO:1	PVCP00000000
MOD1_LR641	Sodium caseinate	USA	4534	56.7	4297	21, CC21	CsakO:1	PVCQ00000000
MOD1_LR643	Soy flour	USA	4500	56.9	4284	73, CC73	CsakO:7	PVDI00000000
MOD1_LR646	Soy flour	USA	4522	56.8	4307	73, CC73	CsakO:7	PVDJ00000000
MOD1_LR688	Org ^c^ flour	USA	4403	57	4169	1, CC1	CsakO:1	PVDF00000000
MOD1_LR702	Powdered infant formula	USA	4648	56.7	4449	21, CC21	CsakO:1	PVDG00000000
MOD1_LR703	Flour	USA	4428	57.1	4180	22	CsakO:2	PVCT00000000
MOD1_LR704	Flour	USA	4404	57	4188	1, CC1	CsakO:1	QHGX00000000
MOD1_LR705	Org. soy powder	USA	4481	56.9	4325	4, CC4	CsakO:2	QHGW00000000
MOD1_LR706	Org. soy powder	USA	4488	56.9	4304	4, CC4	CsakO:2	PVCU00000000
MOD1_LR711	Org. soy powder	USA	4545	56.7	4371	1, CC1	CsakO:1	PVCV00000000
MOD1_LR712	Org. soy powder	USA	4472	56.9	4318	ND ^d^	CsakO:2	PVCW00000000
MOD1_LR713	Environment	USA	4465	57	4266	83, CC83	CsakO:7	PTOX00000000
MOD1_LR714	Org. soy powder	USA	4553	56.7	4360	1, CC1	CsakO:1	PVCX00000000
MOD1_LR721	Org. soy powder	USA	4401	57	4144	12	CsakO:4	PVCY00000000
MOD1_LR723	Org. flour powder	USA	4471	56.9	4266	1, CC1	CsakO:1	PVCZ00000000
MOD1_LR728	Org. soy	USA	4550	56.7	4403	1, CC1	CsakO:1	PVMX00000000
MOD1_LR735	Whey protein	USA	4675	56.6	4474	1, CC1	CsakO:1	PVDH00000000
MOD1_LR736	Whey protein	USA	4679	56.7	4475	1, CC1	CsakO:1	PVDA00000000
MOD1_LR745	Unknown food powder	USA	4598	56.9	4395	4, CC4	CsakO:2	PVMY00000000
MOD1_LR747	Seasoning powder blend ^e^	USA	4675	56.7	4540	13, CC13	CsakO:2	PVDB00000000
MOD1_LR751	Org. soy powder	USA	4436	56.8	4251	4, CC4	CsakO:3	PVDC00000000
MOD1_LR755	Org. soy powder	USA	4557	56.7	4364	1, CC1	CsakO:1	PVDD00000000
MOD1_LR756	Org. flour powder	USA	4577	56.8	4405	13, CC13	CsakO:2	PVDE00000000
MOD1_Jor100	Semolina	Jordan	4362	57	4181	643	CsakO:2	NITS00000000
MOD1_Jor175	Spice	Jordan	4364	56.9	4121	1, CC1	CsakO:1	NITO00000000
MOD1_Jor204	Liquorice	Jordan	4352	57.1	4097	223	CsakO:7	QHGV00000000
MOD1_KW9	Sorghum	Republic of Korea	4610	56.8	4405	143, CC143	ND ^f^	NITF00000000

^a^ Sequence type was determined by uploading genome assemblies to https://pubmlst.org/Cronobacter (last accessed 10 November 2021). ^b^ CC, clonal complex. ^c^ Org., organic. ^d^ The novel MLST for strain MOD1_LR712 needs to be clarified as it matched only 6 of the seven alleles which were indistinguishable from members of the clonal complex 4 (CC4) and its *fusA* allele sequence is also different. This result suggests that this strain possesses a new ST that is phylogenetically related to members of CC4. ^e^ Seasoning powder blend included paprika, onion powder, garlic powder, black pepper, salt, and cayenne pepper. ^f^ Serotyping was determined by following the protocol described by Yan et al. [44]. ND refers to a “not determined” outcome when tested for the presence of a known LPS serotype. Metadata of the 88 plant-origin strains from this study is available following BioSample links at the GenomeTrakr BioProjects: PRJNA258403 and PRJNA613494.

**Table 2:** Antimicrobial resistance genes identified in plant-associated *C. sakazakii* genomes ^a^.

Gene	Sequence Name	Subclass	Strain
*blaCSA-1*	Class C beta-lactamase CSA-1	Cephalothin	Jor20, Jor22 (2 strains) ^b^
*blaCSA-2*	Class C beta-lactamase CSA-2	Cephalothin	LR631, LR632, LR705, LR706, LR712, LR715, LR745, LR751, Jor96, Jor148, Jor154, Jor178 (12 strains) ^c^
*blaCSA*	CSA family class C beta-lactamase	Cephalothin	LR607, LR626, LR627, LR634, LR635, LR636, LR637, LR639, LR640, LR641, LR643, LR646, LR654, LR688, LR702, LR703, LR704, LR707, LR708, LR711, LR713, LR714, LR721, LR722, LR723, LR728, LR733, LR735, LR736, LR747, LR752, LR753, LR755, LR756, LR757, KW1, KW3, KW4, KW9, KW11, KW13, KW18, 1-15, 3-21, 5-17G, 5-20G, 5-21G, O21-13, O21-16, O23mB, O26-1, O26-4, WNTSBCO4, 16MP2184, 16MP2185, 760029, 777122, 788569, AS-13, AS-15, AS-2, AS-4, Jor44, Jor93, Jor100, Jor103, Jor109, Jor146, Jor151, Jor172, Jor173, Jor175, Jor183, Jor204 (74 strains) ^d^
*mcr-9.1*	Phosphoethanolamine-lipid A transferase *mrc-9.1*	Colistin	LR735, LR736, WNTSBOCO4

^a^ Galaxy GenomeTrakr AMRFinder tool used for the identification of antimicrobial resistance (AMR). All 88 FASTA genomes of *C. sakazakii* were scanned with 2 positive controls (p505108-MDR and p505108-NDM), which captured the acquired antimicrobial resistance genes. Raw results and detail are shown in Supplemental Table S2. ^b,c,d^ All 88 *C. sakazakii* genomes were identified as positive for CSA family class C beta-lactamase genes with 100% coverage and 97.33 to 100% identity to reference sequence.

**Table 3:** Summary of the number of genes and percentage of the genes (averages) that are associated with COG functional categories found among the 33 *C. sakazakii* strains evaluated in this study.

Code	Value	% Avg ^a^	Description
A	1	0.0	RNA processing and modification
B	0	0.0	Chromatin structure and dynamics
C	211	5.5	Energy production and conversion
D	45	1.2	Cell cycle control, Cell division, chromosome partitioning
E	293	7.6	Amino acid transport and metabolism
F	100	2.6	Nucleotide transport and metabolism
G	328	8.5	Carbohydrate transport and metabolism
H	140	3.6	Coenzyme transport and metabolism
I	79	2.0	Lipid transport and metabolism
J	180	4.7	Translation, ribosomal structure and biogenesis
K	298	7.7	Transcription
L	183	4.7	Replication, recombination and repair
M	259	6.7	Cell wall/membrane biogenesis
N	67	1.7	Cell motility
O	151	3.9	Posttranslational modification, protein turnover, chaperones
P	247	6.4	Inorganic ion transport and metabolism
Q	45	1.2	Secondary metabolites biosynthesis, transport and catabolism
R	0	0.0	General function prediction only
S	1056	27.3	Function unknown
T	158	4.1	Signal transduction mechanisms
U	66	1.7	Intracellular trafficking and secretion
V	48	1.3	Defense mechanisms
-	0	0.0	Not in COGs

^a^ The number is based on the total number of predicted protein coding genes (3860) in the *C. sakazakii* genomes (average). Total number of genes associated with COG category per individual strain is shown in Supplemental Table S4.

**Table 4:** Prevalence and distribution of pESA3-like incFIB, pESA2-like incFII, and pCTU3/H1-like plasmids and virulence factors harbored on pESA3-like plasmids among the plant-associated *C. sakazakii* isolates.

Total No. of *C. sak* Isolates	pESA3/ pCTU1 (incFIB)	pESA2 /pCTU2(incFII)	pCTU3 /H1	No. of Isolates with the Indicated Plasmidotype (%)										
				*cpa*		T6SS					FHA		Iron Acquisition	
				*cpa* ^a^	Δ*cpa*	Int L	*vgrG*	R end	Int R	ΔT6SS	*fhaB*	ΔFHA	*eitA*	*iucC*
92	92 (100)	5 (6)	20 (30)	91 (99)	0 (0)	91 (99)	50 (54)	56 (60)	29 (31)	0 (0)	19 (21)	66 (72)	92 (100)	92 (100)

^a^*C. sakazakii* strain ATCC 29544^T^ was PCR-negative for the presence of *cpa* which was reported earlier by Franco et al. [45]. Note: This table included four reference strains of *C. sakazakii* (ATCC 29544^T^, BAA-894, GP1999, and H169/1/16) in addition to 88 plant-origin *C. sakazakii* strains. The percentage values of isolates with plasmidotypes are given in parenthesis.

## Data Availability

Data are available upon request.

## Supplementary Materials

The following supporting information can be downloaded at: https://www.mdpi.com/article/10.3390/microorganisms10071396/s1, Figure S1: Emerging lineage groups displaying robust nucleotide divergence of *C. sakazakii* isolates; Figure S2: The first major cluster consisted of 22 ST1, CC1 strains which came from PIF samples (control *C. sakazakii* strain BAA-894), various dried flours, nuts, sodium caseinate, honey powder, and spices. The diversity was represented by two subclades. The second major cluster was made up of five smaller clades each with strains with defined STs such as ST4, CC4; ST31, CC31; ST148, CC16; ST3, CC3; ST17, CC17; ST99, CC99; ST73, CC73; ST64, CC64; ST40, CC40; and ST13, CC13, respectively. There were an additional eleven strains (singletons) possessing STs of ST219, CC155; ST22; ST218; ST93; ST223; ST145; ST136; ST23, CC23; ST198, CC52; and ST12. Interestingly, one ST13, CC13 strain (LR747) clustered with a group of 11 ST4, CC4 strains and a single ST4, CC4 strain (LR745) clustered with eight ST13, CC13 strains. Please refer to the Section 2.6 and Section 3.7 for more details; Table S1: Strain information of *C. sakazakii* isolates (plant-origin foods, production environments, and clinical) used for the phylogenetic microarray and WGS analyses in this study; Table S2: Antibiotic resistance genes identified in 88 plant-associated *C. sakazakii* genomes and 2 positive controls of p505108-MDR (KY978628.1) and p505108-NDM (KY978629.1) by CFSAN’s GalaxyTrakR AMR finder tool; Table S3: Identification of prophage genomic regions within 88 plant-associated *C. sakazakii* using PHASTER bioinformatic tool; Table S4: Number of genes associated with COG category present in each individual plant-associated *C. sakazakii* strains.
