# Supplementary material for: Characterization of Cronobacter sakazakii Strains Originating from Plant-Origin Foods Using Comparative Genomic Analyses and Zebrafish Infectivity Studies
Source: Microorganisms. 2022 Jul 11;10(7):1396. doi: 10.3390/microorganisms10071396 (PMC9319161; doi:10.3390/microorganisms10071396)
Supplement: Supplementary file 1 [file microorganisms-10-01396-s001.zip › S1_Supplement Table S1. Strains _ round3.pdf]

Supplemental Table S1. Strain information of *C. sakazakii* isolates (plant-origin foods, production environments, and clinical) used for the phylogenetic microarray and WGS analyses in this study

Strain	Source	Country	ST[a], CC[b]	Serotype[c]	NCBI Accession no.	Reference
MOD1_LR631	Instant oatmeal	USA	4, CC4	CsakO:2	PTPF00000000	Jang et al., 2018a[1]
MOD1_LR632	Environment	USA	4, CC4	CsakO:2	PTPE00000000	Jang et al., 2018a[1]
MOD1_LR634	Dried cut carrots	USA	148, CC16	CsakO:1	PTPD00000000	Jang et al., 2018a[1]
MOD1_LR635	Dried cut carrots	USA	148, CC16	CsakO:1	PTPC00000000	Jang et al., 2018a[1]
MOD1_LR640	Sodium caseinate	USA	1, CC1	CsakO:1	PTPB00000000	Jang et al., 2018a[1]
MOD1_LR654	Adult cereal	USA	23, CC23	CsakO:2	PTPA00000000	Jang et al., 2018a[1]
MOD1_LR707	Org[d] flour	USA	1, CC1	CsakO:1	PTOZ00000000	Jang et al., 2018a[1]
MOD1_LR708	Org. flour	USA	1, CC1	CsakO:1	PTOY00000000	Jang et al., 2018a[1]
MOD1_LR715	Org. soy	USA	4, CC4	CsakO:2	PTOW00000000	Jang et al., 2018a[1]
MOD1_LR722	Org. soy	USA	1, CC1	CsakO:1	PTOV00000000	Jang et al., 2018a[1]
MOD1_LR733	Org. flour	USA	1, CC1	CsakO:1	PTOU00000000	Jang et al., 2018a[1]
MOD1_LR752	Honey powder	USA	1, CC1	CsakO:1	PTOT00000000	Jang et al., 2018a[1]
MOD1_LR753	Honey powder	USA	31, CC31	CsakO:2	PTOS00000000	Jang et al., 2018a[1]
MOD1_LR757	Org. flour	USA	13, CC13	CsakO:2	PTOR00000000	Jang et al., 2018a[1]
MOD1_1-15	Nuts	South Korea	136	CsakO:2	NITJ00000000	Jang et al., 2018a[1]
MOD1_3-21	Nuts	South Korea	1, CC1	CsakO:1	NITK00000000	Jang et al., 2018a[1]
MOD1_5-17G	Nuts	South Korea	226, CC8	CsakO:1	NITM00000000	Jang et al., 2018a[1]
MOD1_5-20G	Nuts	South Korea	17, CC17	CsakO:2	NITL00000000	Jang et al., 2018a[1]
MOD1_5-21G	Nuts	South Korea	17, CC17	CsakO:2	NIXM00000000	Jang et al., 2018a[1]
MOD1_KW1	Barley	South Korea	93	ND[e]	NITI00000000	Jang et al., 2018a[1]
MOD1_KW4	Dried seaweed	South Korea	73, CC73	ND	NITG00000000	Jang et al., 2018a[1]
MOD1_KW11	Black bean	South Korea	642	CsakO:4	NITE00000000	Jang et al., 2018a[1]
MOD1_KW18	Mushroom	South Korea	156, CC21	CsakO:1	NITC00000000	Jang et al., 2018a[1]
MOD1_777122	Hulled sesame seed	India	198, CC52	CsakO:4	PTOQ00000000	Jang et al., 2018a[1]

MOD1_760029	Whole grain, Corn	Puerto Rico	40, CC40	CsakO:4	PTOP00000000	Jang et al., 2018a[1]
MOD1_16MP002184	Chocolate org. shake	USA	40, CC40	ND	PTOO00000000	Jang et al., 2018a[1]
MOD1_16MP002185	Chocolate org. shake	USA	3, CC3	ND	PTON00000000	Jang et al., 2018a[1]
MOD1_Jor109	Grapes	Jordan	643	CsakO:2	NITQ00000000	Jang et al., 2018a[1]
MOD1_WNTSBCO4	Walnut	USA	1, CC1	ND	PTOM00000000	Jang et al., 2018a[1]
MOD1_Jor173	Unknown spice	Jordan	1, CC1	CsakO:1	PVCG00000000	Jang et al., 2018b[2]
MOD1_Jor146	Liquorice	Jordan	3, CC3	CsakO:2	PVMV00000000	Jang et al., 2018b[2]
MOD1_Jor96	Fennel	Jordan	4, CC4	CsakO:3	PVCE00000000	Jang et al., 2018b[2]
MOD1_Jor148	Unknown spice	Jordan	4, CC4	CsakO:2	PVCF00000000	Jang et al., 2018b[2]
MOD1_Jor154	Unknown spice	Jordan	4, CC4	CsakO:3	NITP00000000	Jang et al., 2018b[2]
MOD1_Jor178	Chamomile	Jordan	4, CC4	CsakO:3	PVBV00000000	Jang et al., 2018b[2]
MOD1_KW13	Dried garlic	South Korea	13, CC13	CsakO:1	NITD00000000	Jang et al., 2018b[2]
MOD1_Jor183	Unknown spice	Jordan	21, CC21	CsakO:1	NITN00000000	Jang et al., 2018b[2]
MOD1_788569	Siberian ginseng, *Eleutherom sentiocosus* Root Powder	China	31, CC31	CsakO:2	PVCL00000000	Jang et al., 2018b[2]
MOD1_KW3	Dried hot pepper	South Korea	40, CC40	CsakO:4	NITH00000000	Jang et al., 2018b[2]
MOD1_AS-2	Allspice	USA	64, CC64	ND	PVCH00000000	Jang et al., 2018b[2]
MOD1_AS-4	Allspice	USA	64, CC64	ND	PVCI00000000	Jang et al., 2018b[2]
MOD1_AS-13	Allspice	USA	64, CC64	ND	PVCJ00000000	Jang et al., 2018b[2]
MOD1_AS-15	Allspice	USA	64, CC64	ND	NCWD00000000	Jang et al., 2018b[2]
MOD1_Jor172	Unknown spice	Jordan	64, CC64	CsakO:2	PVSQ00000000	Jang et al., 2018b[2]
MOD1_O21_16	Oregano	USA	99, CC99	CsakO:1	PVSQ00000000	Jang et al., 2018b[2]
MOD1_O26_1	Oregano	USA	99, CC99	CsakO:1	PVBX00000000	Jang et al., 2018b[2]
MOD1_O21_13	Oregano	USA	219, CC155	CsakO:1	PVBW00000000	Jang et al., 2018b[2]
MOD1_O23mB	Oregano	USA	226, CC8	CsakO:1	PVBZ00000000	Jang et al., 2018b[2]
MOD1_O26_4	Oregano	USA	226, CC8	CsakO:1	PVBY00000000	Jang et al., 2018b[2]
MOD1_Jor20	Unknown spice	Jordan	226, CC8	CsakO:1	PVCA00000000	Jang et al., 2018b[2]
MOD1_Jor22	Chamomile	Jordan	226, CC8	CsakO:1	PVCB00000000	Jang et al., 2018b[2]
MOD1_Jor44	Unknown spice	Jordan	8, CC8	CsakO:1	PVCC00000000	Jang et al., 2018b[2]

MOD1_Jor151	Unknown spice	Jordan	8, CC8	CsakO:1	PVMW00000000	Jang et al., 2018b[2]
MOD1_Jor93	Unknown spice	Jordan	643	CsakO:2	PVCD00000000	Jang et al., 2018b[2]
MOD1_Jor103	Unknown spice	Jordan	643	CsakO:2	NITR00000000	Jang et al., 2018b[2]
GP1999	Tomato plant rhizosphere	Germany	145	ND	NHTW00000000	Chase et al., 2017a[3]
NCTC 8155	Milk powder	UK	4, CC4	CsakO:2	NZ_CP012253	Jaradat et al., 2009[4]
SP291	PIF environment	Ireland	4, CC4	CsakO:2	NC_020260	Power et al. 2013[5]
H169-1-AC	PIF environment	Switzerland	64, CC64	CsakO:2	NHTV00000000	Gopinath et al., 2018[6]
MOD1-H322	PIF product	Switzerland	83, CC83	CsakO:7	MRXM00000000	Chase et al., 2017b[7]
MOD1-H2399	PIF environment, Vacuum cleaner	Switzerland	83, CC83	ND	MRXI00000000	Chase et al., 2017b[7]
MOD1-A31	PIF environment, Floor drain	Switzerland	83, CC83	ND	MRXT00000000	Chase et al., 2017b[7]
Cro2819A3	Mushroom	China	64, CC64	ND	MBSC00000000	Zheng et al. 2017[8]
E772	Milk powder	France	64, CC64	CsakO:2	NHQS00000000	Chase et al., 2017b[7]
E788	Clinical	Ireland	4, CC4	CsakO:2	QWKN00000000	This study
ATCC29004	Clinical	USA	4, CC4	CsakO:2	QWKO00000000	This study
CDC1121_73	Clinical	USA	64, CC64	CsakO:2	MCOD00000000	Chase et al., 2017b[7]
693	Clinical	France	13, CC13	ND	JOLO00000000	Masood et al., 2015[9]
700	Clinical	France	13, CC13	ND	JOLL00000000	Masood et al., 2015[9]
E654	Clinical	Ireland	1, CC1	CsakO:1	NCWF00000000	This study
Csak BAA894	Clinical*	USA	1, CC1	CsakO:1	NC_009778	Kucerova et al., 2010[10]
254N	Clinical	Ireland	1, CC1	CsakO:1	NCWG00000000	This study
Csak29544^{T**}	Clinical	USA	8, CC8	CsakO:1	JMRI00000000	NCBI[11]
HB04	Clinical	China	83, CC83	ND	LGRL00000000	Cui et al. 2017[12]

[a]Sequence type (ST) was determined by uploading genome assemblies to https://pubmlst.org/cronobacter (last accessed 10.13.2021);

[b]CC, clonal complex;

[c]Serotyping was determined by following the protocol described by Yan et al. [44].

[d]Org., organic;

[e]ND refers to a "not determined" finding for the presence of a known LPS serotype.

[*]Pulsed field gel electrophoresis analysis of *C. sakazakii* BAA-894, a PIF isolate was indistinguishable from the clinical isolates that were obtained in 2001 Tennessee outbreak [15].

[**]Csak29544^T is the species type strain.

Supplemental references

1) Jang, H.; Addy, N.; Ewing, L.; Jean-Gilles Beaubrun, J.; Lee , Y.; Woo, J.; Negrete, F.; Finkelstein, S.; Tall, B.D; Lehner, A,.;Eshwar, A.; Gopinath, G.R. Whole-Genome sequences of *Cronobacter sakazakii* isolates obtained from foods of plant origin and dried-food manufacturing environments. *Genome Announc.* **2018,** 6, e00223-18. http://doi:10.1128/genomeA.00223-18
2) Jang, H.; Gopinath, G.R.; Chase, H.R.; Gangiredla, J.; Eshwar, A.; Patel. I.; Addy, N.; Ewing, L.; Beaubrun, J.J.G.; Negrete, F.; Finkelstein, S.; Woo, J.; Lee, Y.; Jaradat, Z.W.; Seo, K.; Fanning, S.; Stephan, R.; Lehner, A.; Tall, B.D. Draft genomes of *Cronobacter sakazakii* strains isolated from dried spices bring unique insights into the epidemiology of plant-associated strains. *Std. Genomics Sciences.* **2018**, 13, 35. https://doi.org/10.1186/s40793-018-0339-6
3) Chase, H.R.; Eberl, L.; Stephan, R.; Jeong, H.; Lee, C.; Finkelstein, S.; Negrete, F.; Gangiredla, J.; Patel, I.; Tall, B.D.; Gopinath, G.R.; Lehner, A. Draft genome sequence of *Cronobacter sakazakii* GP1999, sequence type 145, an epiphytic isolate obtained from the tomato's rhizoplane/rhizosphere continuum. *Genome Announc.* **2017,** 5, e00723-17. https://doi.org/10.1128/genomeA.00723-17.
4) Jaradat, Z.W.; Ababneh, Q.O.; Saadoun, I.M.; Samara, N.A.; Rashdan, A.M. Isolation of *Cronobacter* spp. (formerly *Enterobacter sakazakii*) from infant food, herbs and environmental samples and the subsequent identification and confirmation of the isolates using biochemical, chromogenic assays, PCR and 16S rRNA sequencing. *BMC Microbiol*. **2009**, 9, 225. https://doi.org/10.1186/1471-2180-9-225.
5) Power, K.A.; Yan, Q.; Fox, E.M.; Cooney, S.; Fanning, S. Genome sequence of *Cronobacter sakazakii* SP291, a persistent thermotolerant isolate derived from a factory producing powdered infant formula. *Genome Announc*. **2013**, *1*, e00082–13.
6) Gopinath, G.R.; Chase, H.R.; Gangiredla, J.; Eschwar, A.; Jang, H.; Patel, I.; Negrete, F.; Finkelstein, S.; Park, E.; Chung, T.; et al. Genomic characterization of malonate positive *Cronobacter sakazakii* serotype O:2, sequence type 64 strains, isolated from clinical, food, and environment samples. *Gut Pathog.* **2018**, *10*, 11. https://doi.org/10.1186/s13099-018-0238-9.
7) Zeng, H.; Zhang, J.; Li, C.; Xie, T.; Ling, N.; Wu, Q.; Ye, Y. The driving force of prophages and CRISPR-Cas system in the evolution of *Cronobacter sakazakii*. *Sci. Rep.* **2017**, *7*, 40206.
8) Chase, H.R.; Gopinath, G.R.; Eshwar, A.K.; Stoller, A.; Fricker-Feer, C.; Gangiredla, J.; Patel, I.R.; Cinar, H.N.; Jeong, H.; Lee, C.; Negrete, F.; Finkelstein, S.; Stephan, R.; Tall, B.D.; Lehner, A. Comparative genomic characterization of the highly persistent and potentially virulent *Cronobacter sakazakii* ST83, CC65 strain H322 and other ST83 strains. *Front Microbiol.* **2017**, 8, 1136. http://doi:10.3389/fmicb.2017.01136
9) Masood N, Moore K, Farbos A, Paszkiewicz K, Dickins B, McNally A, Forsythe S. Genomic dissection of the 1994 Cronobacter sakazakii outbreak in a French neonatal intensive care unit. BMC Genomics. 2015 Oct 5;16:750. doi: 10.1186/s12864-015-1961-y.

10) Kucerova, E.; Clifton, S.W.; Xia, X.Q.; et al. Genome sequence of *Cronobacter sakazakii* BAA-894 and comparative genomic hybridization analysis with other *Cronobacter* species. *PLoS One.* **2010** 5, e9556. http://doi:10.1371/journal.pone.0009556.
11) NCBI* - Search with Accession No. at https://www.ncbi.nlm.nih.gov/nuccore/
12) Cui, J.H.; Yu, B.; Xiang, Y.; Zhang, Z.; Zhang, T.; Zeng, Y.C.; Cui, Z.G.; Huo, X.X. Two cases of multi-antibiotic resistant *Cronobacter* spp. infections of infants in China. *Biomed. Environ. Sci*. **2017**, *30*, 601–605.
