# Supplementary material for: Characterization of Cronobacter sakazakii Strains Originating from Plant-Origin Foods Using Comparative Genomic Analyses and Zebrafish Infectivity Studies
Source: Microorganisms. 2022 Jul 11;10(7):1396. doi: 10.3390/microorganisms10071396 (PMC9319161; doi:10.3390/microorganisms10071396)
Supplement: Supplementary file 1 [file microorganisms-10-01396-s001.zip › Supplemental Figures revision round 2 final.pdf]

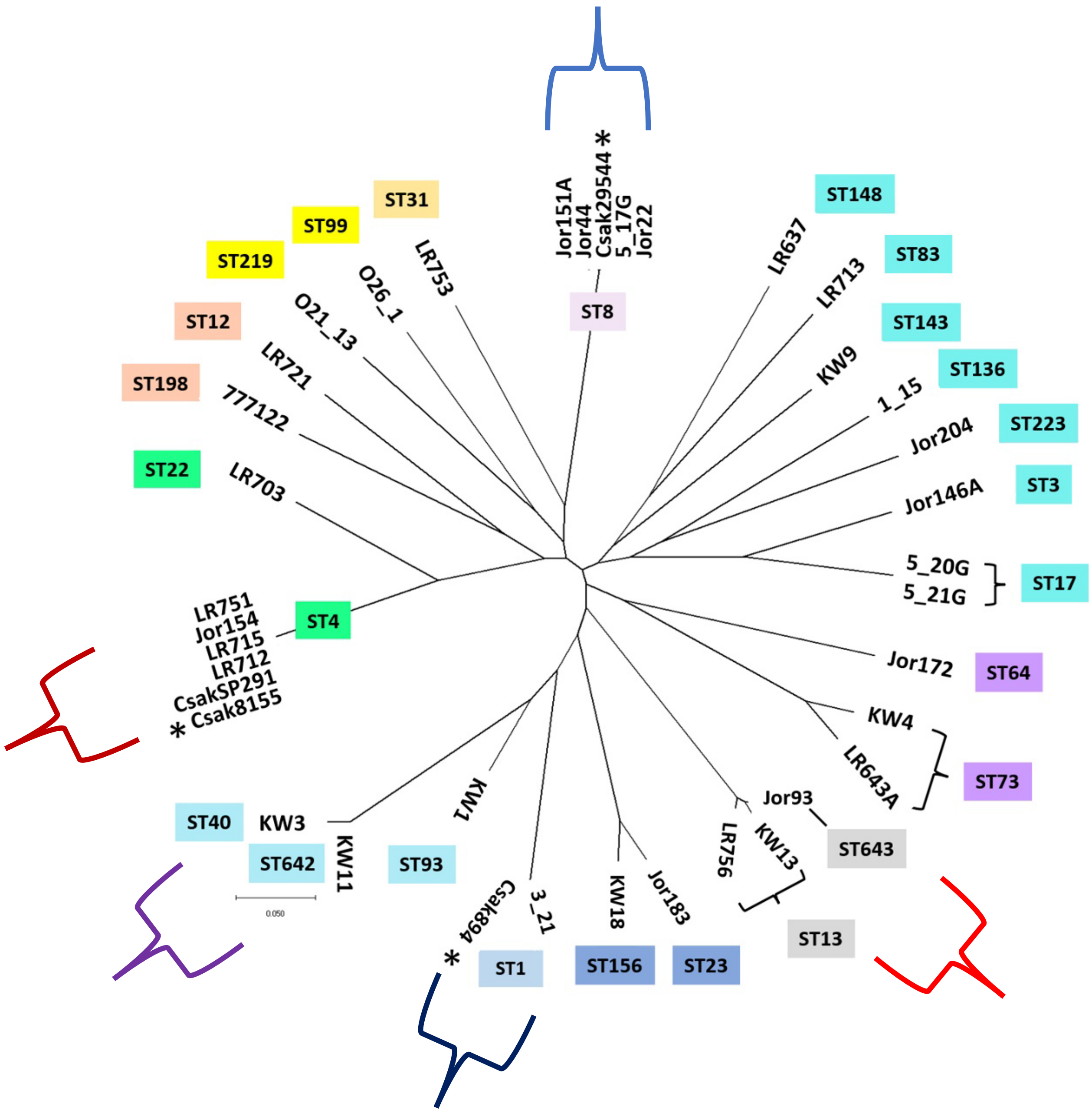

Supplemental Figure S1. Emerging lineage groups displaying robust nucleotide divergence of *C. sakazakii* isolates. Twenty-five selected *C. sakazakii* isolates were clustered based on a sub-set of allele matrix generated as described. Emerging nucleotide divergence was these representative isolates clustered into five 'lineage-groups' (demarcated by different colored additional mark-ups) consisting of 1 or more STs. STs of clinical isolates have an asterisk and result in a heterogeneous mixture of clinical and environmental strains. Some lineage groups with just clinical isolates appear to be distinct in their grouping. Bar marker represents 0.05 base substitutions per site. Lineage groups consisting of 2 or more STs were determined only based on shared ST allelic profiles for this illustration. A deeper comparison of core-gene allelic information may shed light on any shared micro-evolutionary processes between these and other co-clusters suggesting

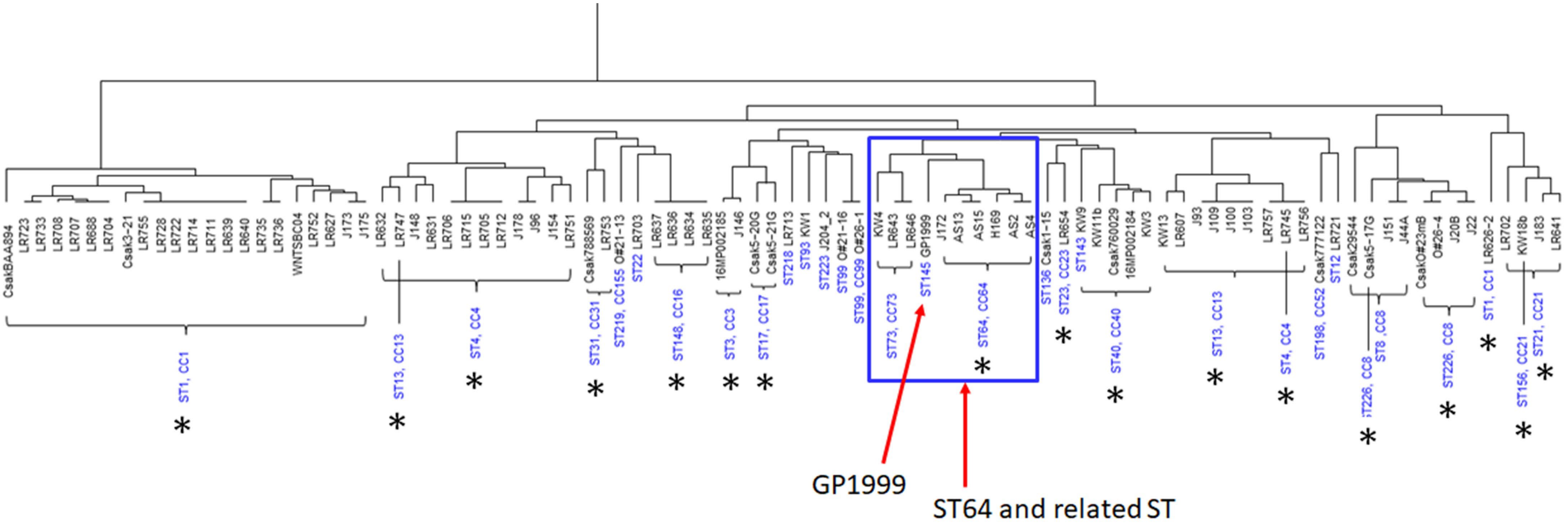

Supplemental Figure S2. The first major cluster consisted of 22 ST1, CC1 strains which came from PIF samples (control *C. sakazakii* strain BAA-894), various dried flours, nuts, sodium caseinate, honey powder, and spices. The diversity was represented by two subclades. The second major cluster was made up of five smaller clades each with strains with defined STs such as ST4, CC4; ST31, CC31; ST148, CC16; ST3, CC3; ST17, CC17; ST99, CC99; ST73, CC73; ST64, CC64; ST40, CC40; and ST13, CC13, respectively. There were an additional eleven strains (singletons) possessing STs of ST219, CC155; ST22; ST218; ST93; ST223; ST145; ST136; ST23, CC23; ST198, CC52; and ST12. Interestingly, one ST13, CC13 strain (LR747) clustered with a group of 11 ST4, CC4 strains and a single ST4, CC4 strain (LR745) clustered with eight ST13, CC13 strains. Please refer to the sections 2.6 and 3.7 for more details.
